# Dynamic stability and stepping strategies of young healthy adults walking on an oscillating treadmill

**DOI:** 10.1371/journal.pone.0212207

**Published:** 2019-02-13

**Authors:** Tanya Onushko, Timothy Boerger, Jacob Van Dehy, Brian D. Schmit

**Affiliations:** 1 Department of Biomedical Engineering, Marquette University, Milwaukee, WI, United States of America; 2 Department of Physical Therapy, Marquette University, Milwaukee, WI, United States of America; 3 Leidos, Inc. Reston, VA, United States of America; University of Rochester, UNITED STATES

## Abstract

Understanding how people modify their stepping to maintain gait stability may provide information on fall risk and help to understand strategies used to reduce loss of balance. The purpose of this study was to identify the stepping strategies healthy young individuals select to maintain balance while walking on a destabilizing surface in various directions. A treadmill mounted on top of a 6 degree-of-freedom motion base was used to generate support surface oscillations in different degrees of freedom and amplitudes. Fifteen healthy young adults (21.3 ± 1.4 years) walked at self-selected speeds while continuous sinusoidal oscillations were imposed to the support surface in a one degree of freedom: rotation or translation in the mediolateral (ML) direction and rotation or translation in the anteroposterior (AP) direction, with each condition repeated at three different amplitudes. We compared step width, length, and frequency and the mean and variability of margin of stability (MoS) during each experimental walking condition with a control condition, in which the support surface was stationary. Subjects chose a common strategy of increasing step width (*p* < 0.001) and decreasing step length (*p* = 0.008) while increasing mediolateral MoS (*p* < 0.001), particularly during oscillations that challenged frontal plane control, with rotations of the walking surface producing the greatest changes to stepping.

## Introduction

A better understanding of the adjustments in spatiotemporal gait parameters individuals make to maintain stable gait on different walking surfaces may provide information on fall risk. Walking in challenging environments can increase the risk and concerns for falling causing individuals to adopt a more cautious gait pattern [1–4]. When gait stability is challenged, such as transitioning between different surfaces [5, 6] or walking on irregular surfaces [7–10], individuals will adjust their foot placement to increase stability and decrease fall risk. During daily activities, individuals encounter a wide variety of environments that challenge stability and stepping strategies could vary depending on size and direction of the destabilizing perturbation. Determining how people adjust foot placement while walking in a broader range of destabilizing environments is necessary to understand fundamental stepping strategies (i.e. spatiotemporal gait parameters) used to reduce the loss of balance.

Maintaining mediolateral (ML) stability during gait is an active control process. It requires the center of mass (CoM) motion (i.e. velocity and position) stays within the base of support (BoS). Based on theoretical models of walking, adjusting foot placement to control the CoM is an efficient technique for modifying balance while walking [11]. Healthy adults take shorter, quicker, and wider steps to increase the BoS and limit CoM motion when walking in an unstable environment [10, 12, 13]; a strategy that appears to be an efficient technique for stabilizing gait based on theoretical models of walking [14].

Dynamic balance can be assessed using the margin of stability (MoS). The MoS takes into consideration the CoM position and velocity with respect to the BoS, such that increasing the distance between the CoM and BoS theoretically increases dynamic stability [11]. In response to balance perturbations, healthy adults will effectively adjust their stepping to maintain or increase the MoS [10, 12, 15–17]. For example, when walking on a self-paced treadmill, healthy adults chose to alter their step parameters (walking with wider, shorter and more frequent steps) rather than alter gait speed in order to increase the ML MoS [12]. Previous studies have examined different factors that influence MoS, such as visual perturbations [10], perturbation intensity [12], or direction [17]. Yet, no study has evaluated how continuous rotations compared to translations of the walking surface affects spatiotemporal gait parameters and ML dynamic stability.

In this study, we examined changes in spatiotemporal gait parameters and ML dynamic stability in response to sinusoidal oscillations of different amplitude, type, and direction imposed to the walking surface. We hypothesized that participants would walk with wider, shorter and quicker steps, increased ML MoS, and MoS variability for all imposed oscillations. Specifically, we hypothesized that these changes: 1) would be the largest during ML compared to anteroposterior (AP) oscillations, 2) would be largest during rotations compared to translations of the walking surface, and 3) would scale with increasing oscillation amplitude.

## Methods

### Subjects

We recruited fifteen young adults (8 males) with average age, height and weight of 21.3 (SD 1.4) years, 1.7 (SD 0.1) m and 68.8 (SD 10.7) kg, respectively. Informed consent was obtained from all participants prior to participating. All experimental procedures were approved by the Institutional Review Board of Marquette University.

### Equipment

**Fig 1:**
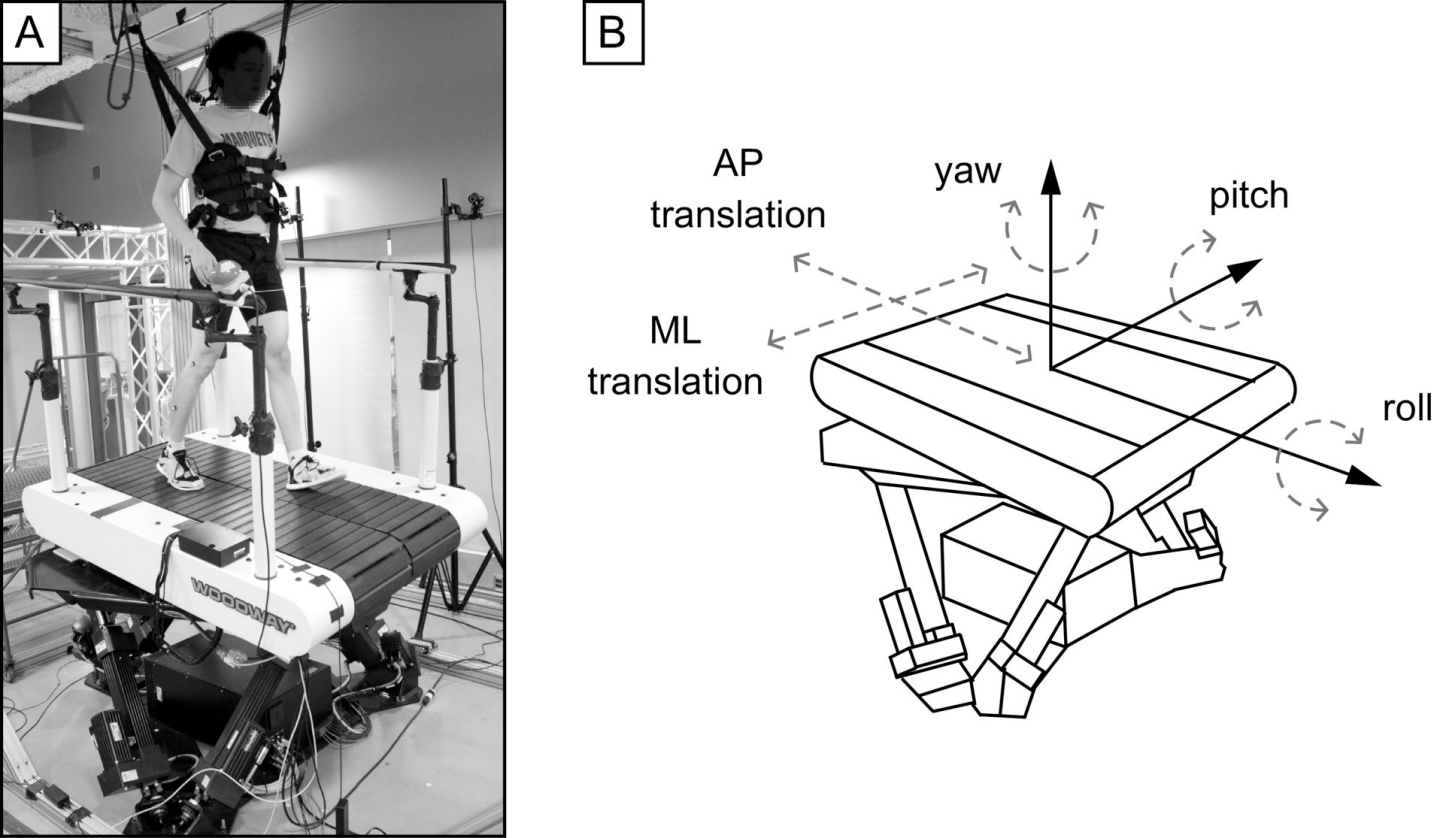
Experimental equipment. (A) Subjects walked on a treadmill mounted on top of a 6 degree-of-freedom motion base. (B) Examples of the degrees of freedom the motion base can move.

Subjects walked on a custom-designed treadmill system (Fig 1A). A Woodway treadmill (Woodway USA Inc., Waukesha, WI, USA) was mounted on a six-axis motion base (Moog Inc., East Aurora, NY, USA) allowing for movement in 6 degrees-of-freedom (Fig 1B). A custom-written LabVIEW program (National Instruments Corp., Austin, TX, USA) was used to control the oscillations of the motion base system. Subjects wore a safety harness during all walking trials, which did not provide weight support.

Kinematic data were collected at 120 Hz from a 14-camera infrared motion capture system (NaturalPoint Inc., Corvallis, OR, USA). Subjects wore reflective markers attached to anatomical landmarks of the anterior superior iliac spine bilaterally, sacrum, greater trochanters, medial and lateral knee joints, medial and lateral ankle malleoli, and first and fifth metatarsal heads bilaterally. The locations of the anatomical landmarks were found by manual palpation. Marker clusters, consisting of 3 markers attached to plastic plates, were strapped around the thighs and shanks, and taped to the heels of the shoes.

### Experimental protocol

#### Familiarization

Prior to beginning the experiment, subjects were familiarized with the equipment. We incrementally increased belt speed until the subject self-reported a comfortable walking speed (mean 0.78 ±0.19 m/s). This speed was used for the experimental trials. Subjects then completed a control walking trial, in which they walked at their comfortable pace for 80 seconds when the motion base was stationary. This walking trial served as our control condition to which experimental trials were compared.

#### Experimental trials

The protocol consisted of thirteen experimental conditions. For twelve conditions, we imposed continuous sinusoidal oscillations (0.12 Hz) to the treadmill in one degree-of-freedom (Fig 1B): Pitch, Roll, ML and AP. We tested a low, medium and high amplitude for each degree-of-freedom: ±5, ±10, and ±15 degrees for Pitch and Roll oscillations, and ±8, ±16.5 and ±25 cm for ML and AP oscillations. The motion base velocity and acceleration are provided in Table 1. Briefly, the same velocity and acceleration were applied during the ML and AP translation trials, and the same rotational velocity and acceleration were applied to the Roll and Pitch trials. Slower accelerations/velocities were selected in order to create challenging, yet achievable walking conditions. The thirteenth condition was a combination of Roll, Pitch and Yaw (RPY) oscillations with offset frequencies (0.15, 0.16, 0.17 Hz, respectively) at an amplitude of ±8 degrees. Subjects walked for 80 seconds per trial and were given 60 seconds rest between trials. Experimental conditions were presented in random order. All subjects were able to walk during the experimental trials without falling and without holding onto handrails.

**Table 1:** Velocity and acceleration of the motion base during the single degree-of-freedom sinusoidal oscillation trials.

**Translation Trials**	**Peak Velocity (m/s)**	**Acceleration (m/s^2^)**
8 cm	0.0418	0.0316
16.5 cm	0.0841	0.0634
25 cm	0.1263	0.0952
**Rotation Trials**	**Peak Velocity (°/s)**	**Peak Acceleration (°/s^2^)**
5 deg	3.6	2.7
10 deg	7.3	5.5
15 deg	10.9	8.2

### Data analysis

#### Kinematic and spatiotemporal parameters

Kinematic data were low-pass filtered using a 4^th^ order Butterworth filter with a cut-off frequency of 6 Hz using Visual 3D (C-Motion Inc., Rockville, MD, USA). We used a local coordinate system to define model segments for the pelvis, thighs, shanks and feet. Kinematic data were then used to automatically approximate heel strikes, which were defined as times when the distance between the pelvis and heel markers was maximum [18], and the events were verified by visual inspection. Step width was calculated as ML distance between heel markers at moment of heel-contact. We approximated step length as the AP distance between heel markers at moment of heel-contact. Both step width and length were normalized to the subject’s leg length. During Roll, Pitch and RPY trials, step width and length were calculated using local coordinates with respect to the motion base (i.e. the angle of the base was taken into account). Step frequency was calculated as the average of inverse step duration for each step. These analyses were performed for the final 60 seconds of each trial, and were conducted in Matlab (The Mathworks, Natick, MA, USA).

#### Dynamic stability

We calculated the ML margin of stability (MoS_ML_) to quantify dynamic stability in the ML direction using previous methods [11, 13]. The MoS_ML_ is the minimum difference between the extrapolated center of mass (XCoM) and BoS (MoS_ML_ = BoS – XCoM_ML_) for each step. The lateral malleolus marker defined the lateral boundary of the BoS. The XCoM was calculated using:

$$\mathrm{XCoM}=\mathrm{CoM}+C\dot{o}M\sqrt{\left(l/g\right)}$$

where CoM and $C\dot{o}M$ are position and velocity of CoM, respectively, *g* is acceleration due to gravity, and *l* is maximum height of the estimated CoM, which was calculated as 1.34 times trochanteric height. The CoM position was estimated using the pelvic segment [19]. During experimental trials, we corrected the MoS by subtracting the velocity factor of treadmill movement, ${\dot{V}}_{motion\;base}\sqrt{\left(l/g\right)}$ from XCoM, where ${\dot{V}}_{motion\;base}$ was either ML or AP linear or tangential velocity of the treadmill [20]. This maintained consistency when comparing the MoS among trials. We calculated the mean and variability (coefficient of variation (CoV)) of the MoS_ML_ during the final 60 seconds of each trial. Increasing MoS_ML_ indicates the XCoM is within the BoS increasing dynamic stability. Decreasing or negative MoS_ML_ indicates the CoM is nearing or outside of the BoS. The MoS variability was used as an indicator of fall risk–increasing MoS variability suggests increased risk of falling [21].

### Statistics

A one-way repeated measures ANOVA was conducted to compare the effect of walking surface oscillation on step frequency, step width, and step length and mean MoS_ML_ and CoV of MoS_ML_. The Greenhouse-Geisser correction factor was used when Mauchly’s test of sphericity was violated. When a significant effect of walking surface oscillation was found, simple contrasts were conducted to test whether the dynamic walking condition differed from the control walking condition. We also conducted a multi-factor repeated measures ANOVA to compare oscillation Type (Rotation, Translation), Direction (AP, ML) and Amplitude (low, medium, high) on the dependent variables. For this analysis, we excluded the Control and RPY conditions. All statistics were performed using IBM SPSS Statistics 21.0 (IBM, New York, NY, USA). The α level was set at 0.05 and a Bonferroni correction (α = 0.0038) was applied for post-hoc multiple comparisons. Data are reported as means (SD). Only significant main effects and contrasts are presented, unless otherwise noted.

## Results

**Fig 2:**
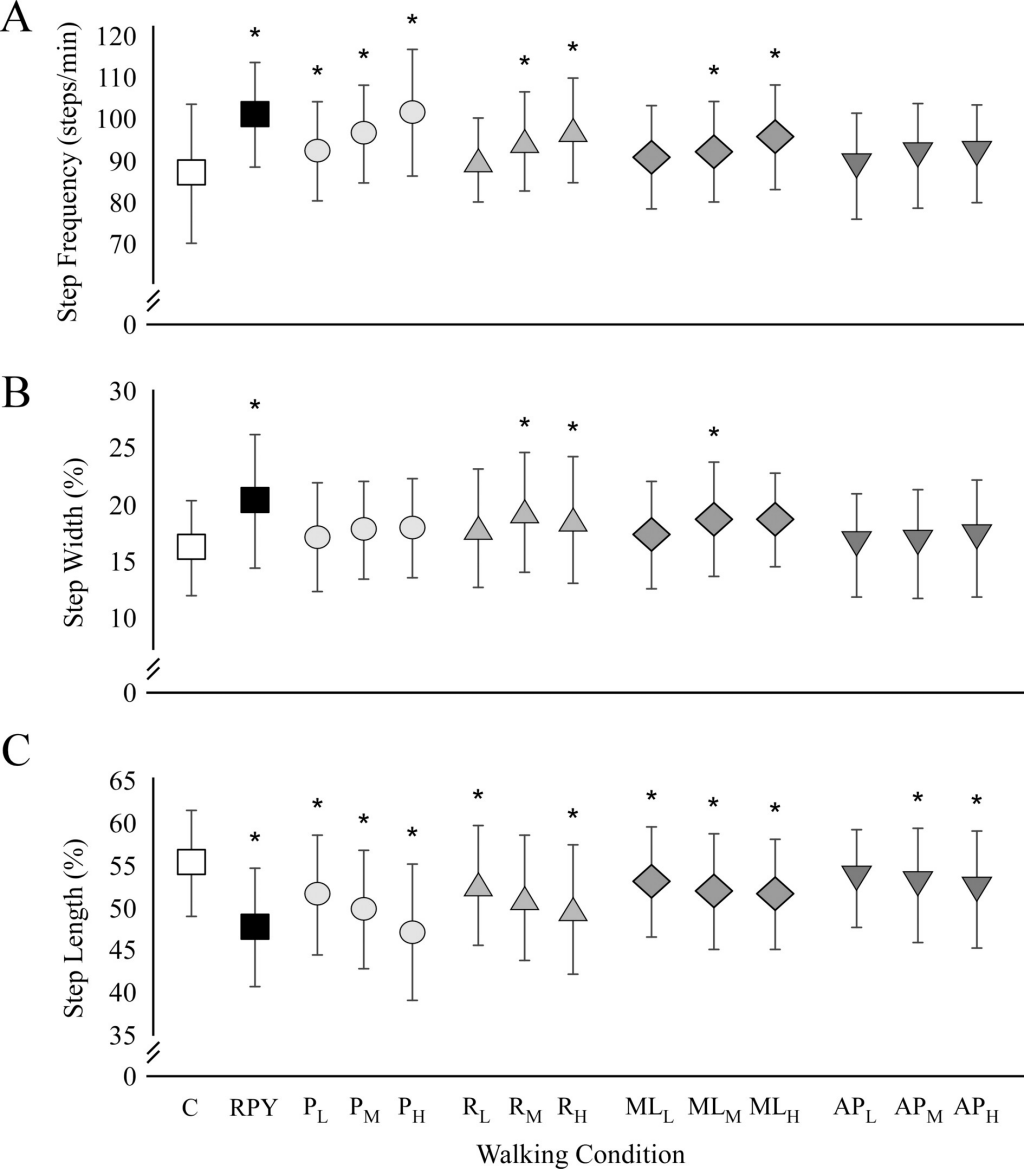
Step parameters. Step frequency (A), step width (B) and step length (C) for all walking conditions are shown for following conditions: control walking condition (C), roll, pitch, yaw combination (RPY), pitch (P_L_, P_M_, P_H_), roll (R_L_, R_M_, R_H_), mediolateral (ML_L_, ML_M_, ML_H_) and anteroposterior (AP_L_, AP_M_, AP_H_) oscillations at low (L), medium (M) and high (H) amplitudes. Asterisks represent significant difference (*p* < 0.05) with respect to the control condition.

Subjects made significant changes to foot placement during walking surface oscillations (Fig 2). We observed a main effect of walking surface oscillation for step frequency (F_13,182_ = 8.66, *p* < 0.001, η_p_^2^ = 0.382), step width (F_13,182_ = 16.24, *p* < 0.001, η_p_^2^ = 0.537), and step length (F_13,182_ = 32.46, *p* = 0.008, η_p_^2^ = 0.699). Subjects took quicker steps during RPY (*p* = 0.017), Pitch (low: *p* = 0.001; medium: *p* < 0.001; high: *p* = 0.013), Roll (medium: *p* = 0.002; high: *p* = 0.001), and ML oscillations (low: *p* = 0.001; medium: *p* < 0.001. respectively) compared to control walking. Subjects also increased step width for RPY (*p* < 0.001), Roll (medium: *p* < 0.001; high: *p* = 0.001), and ML oscillations (medium: *p* = 0.003). For all oscillating conditions, subjects walked with significantly shorter steps compared with the control condition (*p* < 0.001 for all comparisons, except: medium Roll, *p* = 0.007 and low AP, *p* = 0.007).

**Fig 3:**
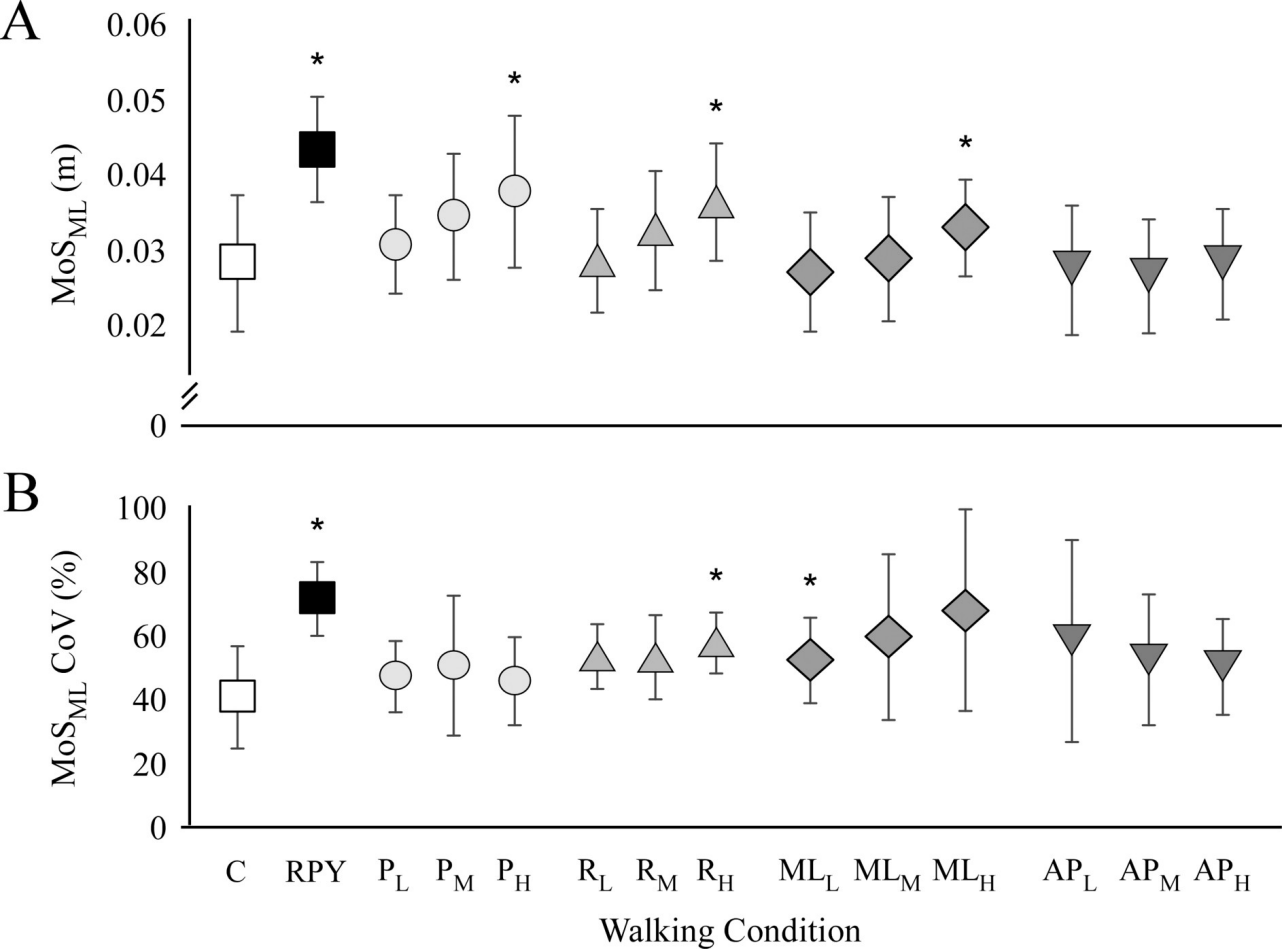
Dynamic stability. The mean MoS_ML_ (A) and the coefficient of variation of MoS_ML_ (B) are shown for following conditions: control walking condition (C), roll, pitch, yaw combination (RPY), pitch (P_L_, P_M_, P_H_), roll (R_L_, R_M_, R_H_), mediolateral (ML_L_, ML_M_, ML_H_) and anteroposterior (AP_L_, AP_M_, AP_H_) oscillations at low (L), medium (M) and high (H) amplitudes. Asterisks represent significant difference (*p* < 0.05) with respect to the control condition.

Subjects walked with larger MoS_ML_ during the oscillations than the control condition (F_13,182_ = 15.2, *p* < 0.001, η_p_^2^ = 0.521; Fig 3A). Simple contrasts revealed that MoS_ML_ was greater for RPY, high Pitch, Roll and ML oscillations compared with the control condition (*p* < 0.001, *p* = 0.001, *p* = 0.001, *p* < 0.001, respectively). Subjects also exhibited greater MoS_ML_ variability during walking surface oscillations (Fig 3B). We found a main effect of walking surface oscillation on MoS_ML_ (F_13,128_ = 4.62, *p* = 0.006, η_p_^2^ = 0.213) variability. Subjects walked with greater MoS_ML_ variability during RPY (*p* < 0.001), high Roll (*p* < 0.001), low ML (*p* < 0.001) and medium (*p* = 0.004) and high (*p* = 0.009) AP oscillations.

We also compared Type (Rotation vs. Translation), Direction (ML vs. AP) and Amplitude (low, medium and high) on stepping strategies and dynamic stability. The statistical results are reported in Table 2. We observed a main effect of Type and Amplitude for step width, length and frequency (*p* < 0.05). Subjects walked with wider, shorter and quicker steps during rotating oscillations (Pitch and Roll) compared to translating oscillations (ML and AP). These parameters also scaled with the oscillation amplitude. High amplitude oscillations resulted in the widest, shortest and quickest steps, and vice versa. We only observed a main effect of Direction on the step width. Subjects took wider steps during the ML directions (Roll and ML) compared to the AP directions (Pitch and AP). The interaction of Type x Direction and Type x Amplitude were significant for step length and frequency only. Post-hoc comparisons for Type x Direction interaction showed that subjects walked with shorter and quicker steps for the rotation oscillations compared to the translation oscillations, particularly for Pitch. Similarly, post-hoc comparisons for Type x Amplitude interaction showed subjects walked with shorter and quicker steps during rotation oscillations compared to translation oscillations at each amplitude (*p* < 0.05 for each comparison).

**Table 2:** Statistical results.

Dependent Measure	Type	Direction	Amplitude	Type x Direction	Type x Amplitude	Direction x Amplitude
Step width	F = 5.2 ***p* = 0.038** η_p_^2^ = 0.27	F = 13.3 ***p* = 0.003** η_p_^2^ = 0.48	F = 7.8 ***p* = 0.002** η_p_^2^ = 0.36	F = 0.95 *p* = 0.347 η_p_^2^ = 0.06	F = 0.27 *p* = 0.769 η_p_^2^ = 0.02	F = 1.89 *p* = 0.169 η_p_^2^ = 0.12
Step length	F = 28.4 ***p* < 0.001** η_p_^2^ = 0.67	F = 3.36 *p* = 0.088 η_p_^2^ = 0.19	F = 58.7 ***p* < 0.001** η_p_^2^ = 0.81	F = 32.2 ***p* < 0.001** η_p_^2^ = 0.72	F = 17.8 ***p* < 0.001** η_p_^2^ = 0.56	F = 1.4 *p* = 0.270 η_p_^2^ = 0.09
Step frequency	F = 17.0 ***p* = 0.001** η_p_^2^ = 0.55	F = 2.59 *p* = 0.130 η_p_^2^ = 0.16	F = 25.9 ***p* < 0.001** η_p_^2^ = 0.65	F = 16.8 ***p* = 0.001** η_p_^2^ = 0.55	F = 8.1 ***p* = 0.002** η_p_^2^ = 0.37	F = 0.02 *p* = 0.979 η_p_^2^ = 0.01
MoS_ML_	F = 32.4 ***p* < 0.001** η_p_^2^ = 0.70	F = 0.04 *p* = 0.837 η_p_^2^ = 0.01	F = 13.4 ***p* < 0.001** η_p_^2^ = 0.49	F = 18.3 ***p* = 0.001** η_p_^2^ = 0.57	F = 7.4 ***p* = 0.003** η_p_^2^ = 0.35	F = 1.7 *p* = 0.209 η_p_^2^ = 0.11

Significance accepted at *p* < 0.05 and are indicated in bold font.

For dynamic stability, we observed significant main effects of Type and Amplitude on the MoS_ML_. Subjects walked with greater MoS_ML_ during rotation oscillations (Pitch and Roll) compared to translation oscillations. The MoS_ML_ also scaled with the oscillation amplitude–the MoS_ML_ increased with increasing oscillation amplitude. We observed significant interaction effects between Type and Direction on MoS. Post-hoc comparisons showed a larger MoS_ML_ during rotation in the AP and ML-directions compared to translation in AP or ML (*p* < 0.05 for both comparisons). Lastly, we also observed a significant interaction between Type and Amplitude on the MoS. Post-hoc comparisons showed a larger MoS_ML_ during rotation oscillations at each amplitude compared to the translation oscillations (*p* < 0.05 for all comparisons).

## Discussion

The aim of this study was to determine how continuous oscillations of the walking surface at differing amplitudes, types and directions affects stepping strategies and dynamic balance in healthy young adults. Compared to level walking, healthy adults took wider steps particularly for trials in the ML direction (Roll and ML translation). The oscillation amplitude and type (rotation trials Roll and Pitch) applied to the walking surface resulted in an increase in step frequency and decrease in step lengths, with rotations applied in the AP direction (Pitch) resulting in the greatest change in step length and frequency. Dynamic stability was also dependent on the type of movement. Rotation oscillations caused the largest increase in ML MoS compared to the translation oscillations.

As reported in similar studies, the observed stepping adjustments in response to different walking surface oscillations suggest that this is a natural strategy to increase dynamic stability, and potentially reduce loss of balance [10, 12, 13, 15, 17, 22]. From the current study, the increase in ML MoS and its relationship to loss of balance may be interpreted in several ways. First, an increased dynamic stability during the oscillating conditions suggests the participants were more stable, and thus, less likely to fall, especially during the more challenging walking conditions (i.e. RPY, Roll_H_). Participants walked with wider steps to increase the distance between the XCoM and BoS thereby increasing ML MoS compared to the control walking condition. A second interpretation is that young healthy adults walked with wider steps to increase ML MoS, but their risk for falls may still be the same as the control walking condition. In this latter scenario, the walking environment itself imposes a challenge to balance and healthy adults naturally will adjust their stepping to maintain dynamic stability.

Another potential interpretation may be that subjects favored control of frontal plane balance over sagittal plane balance. Shortening step length can also be regarded as a strategy to decrease risk of falling when walking in unstable conditions [23]. For example, shortening step length will move the CoM near or even in front of the BoS (i.e. in front of the leading foot), which will reduce a chance of backwards loss of balance but at the cost of potentially inducing a forward loss of balance [12, 24, 25]. In the current study, we observed decreased step length, with the shortest step length occurring in the Rotation conditions at high amplitude. Together with taking wider steps, taking shorter steps could imply that subjects are still at risk for loss of balance in the sagittal plane in order to favor better frontal plane control. However, we cannot verify this from the current data because the XCoM model for AP stability does not consider angular momentum, which can influence AP stability, misrepresenting true stability in the sagittal plane during up/down hill walking [26]. Furthermore, the changes in knee and hip angles during the Pitch trials affect joint power and stiffness, which violates the assumption that the inverted pendulum model is a passive system [27]. Further work is needed to define the relationship between ML MoS, AP MoS and fall risk when walking on challenging environments.

One major finding is that subjects made greater adjustments to foot placement during rotating oscillations. We observed increased step width during the Roll oscillations, and decreased step length and increased step frequency during the Pitch oscillations. Furthermore, these stepping adjustments were more pronounced during the rotating oscillations compared to the translation oscillations for each oscillation amplitude (i.e. low, medium and high). Sloped walking requires a person to alter their ground reaction forces, and therefore joint moments, to maintain or increase stability. For example, during side-sloped walking, subjects will decrease hip joint moments but increase moment about the knee and ankle to keep the CoM stable [28]. While our results are similar to previous studies investigating sloped walking [29–31], it is important to note that we cannot make direct comparisons of the oscillation amplitudes between rotating and translating conditions– 15 degrees of rotation may not have the same influence on gait parameters and dynamic stability as 9.8 cm of translation. However, from observation, subjects largely walked in the center of the treadmill, near the rotational axes. This implies this area would have the lowest rotational velocity and acceleration imposed to the person from the motion base.

Walking under continuous sinusoidal oscillations of the walking surface increased MoS_ML_ variability. The observed increase in MoS_ML_ variability may indicate that imposing sinusoidal oscillations of the walking surface in different directions increases the risk for falling for healthy young adults [21]. While a greater MoS when walking can be considered an indicator of dynamic stability [11], MoS variability may be related to how well people control CoM and foot placement when stability is challenged [16, 32]. In our study, subjects maintained sufficient MoS_ML_ when walking during the oscillations, even though they exhibited greater MoS_ML_ variability. This could suggest that they were able to quickly adjust their gait and keep their balance while walking in a destabilizing environment. Conversely, sinusoidal oscillations of the walking surface could have increased MoS variability while participants remained dynamically stable. For example, walking on a laterally oscillating treadmill, healthy adults were shown to alter their stepping at particular motions of the walking surface resulting in increased step width variability [21].

## Methodological considerations

Sinusoidal oscillations created a predictable walking surface that could encourage subjects to time their stepping with the oscillations (i.e. to entrain). Two factors, however, reduce the likelihood of entrainment. First, the average step frequency (1.6 Hz for the control walking condition) was much faster than the imposed sinusoidal frequency (0.12 Hz). Previous studies have shown that dynamic entrainment largely occurs if the period of the perturbation is close to the individual’s preferred cadence [33–35]. Second, rhythmic cueing reduces variability of stepping [36, 37] and we observed increasing levels of stepping variability during the destabilizing conditions.

Walking on elevated surfaces can increase postural threat and raise concern for falling leading to altered gait [38–41]. Walking on elevated surfaces increases the time spent in double support phase, reduces step length, velocity and cadence [38–40]. In our study, subjects walked ~100 cm above the ground. Walking at this height could have increased their fear, causing subjects to walk with a “cautious” gait pattern, and may explain why we observed a slower than normal walking speed for healthy young adults. Despite this, our subjects continued to exhibit changes in stepping similar to previous studies [10, 12, 13].

### Clinical implications

These results enhance our knowledge on how healthy individuals modify their gait in challenging walking conditions and are useful for future studies investigating dynamic balance in balance-impaired individuals. Among clinical populations, walking over uneven terrain increases fall risk and limits mobility [8, 9, 16]. Quantifying dynamic balance of high-fall risk individuals in challenging walking conditions can tell us what factors are important in managing fall risk and how to address rehabilitation concerns. Findings from our study can be used to compare how balance-impaired individuals compensate for the same walking conditions or whether increasing the challenge imposed to balance affects balance-impaired individuals similarly.

## Conclusion

This study demonstrates that healthy young adults adjust their gait pattern to increase dynamic stability when walking over destabilizing surfaces. Subjects took quicker, shorter and wider steps to increase dynamic stability when oscillations were imposed to the walking surface and were largely influenced by rotations and amplitude of oscillation. These results suggest that healthy young adults use a generalized gait strategy to maintain dynamic stability when their balance is challenged.

## Supporting information

S1 Data (XLSX)
